# Enceladus: First Observed Primordial Soup Could Arbitrate Origin-of-Life Debate

**DOI:** 10.1089/ast.2019.2029

**Published:** 2019-10-03

**Authors:** Amit Kahana, Philippe Schmitt-Kopplin, Doron Lancet

**Affiliations:** ^1^ Department of Molecular Genetics, the Weizmann Institute of Science, Rehovot, Israel.; ^2^ Helmholtz Zentrum Muenchen, Research Unit Analytical BioGeoChemistry, Neuherberg, Germany.; ^3^ Technische Universität München, Chair of Analytical Food Chemistry, Freising-Weihenstephan, Germany.

**Keywords:** Enceladus, Lipid First model, Prebiotic chemistry, Carbonaceous chondrite, Origin of life, Mutual catalysis

## Abstract

A recent breakthrough publication has reported complex organic molecules in the plumes emanating from the subglacial water ocean of Saturn's moon Enceladus (Postberg *et al.,* 2018, *Nature* 558:564–568). Based on detailed chemical scrutiny, the authors invoke primordial or endogenously synthesized carbon-rich monomers (<200 u) and polymers (up to 8000 u). This appears to represent the first reported extraterrestrial organics-rich water body, a conceivable milieu for early steps in life's origin (“prebiotic soup”). One may ask which origin-of-life scenario appears more consistent with the reported molecular configurations on Enceladus. The observed monomeric organics are carbon-rich unsaturated molecules, vastly different from present-day metabolites, amino acids, and nucleotide bases, but quite chemically akin to simple lipids. The organic polymers are proposed to resemble terrestrial insoluble kerogens and humic substances, as well as refractory organic macromolecules found in carbonaceous chondritic meteorites. The authors posit that such polymers, upon long-term hydrous interactions, might break down to micelle-forming amphiphiles. In support of this, published detailed analyses of the Murchison chondrite are dominated by an immense diversity of likely amphiphilic monomers. Our specific quantitative model for compositionally reproducing lipid micelles is amphiphile-based and benefits from a pronounced organic diversity. It thus contrasts with other origin models, which require the presence of very specific building blocks and are expected to be hindered by excess of irrelevant compounds. Thus, the Enceladus finds support the possibility of a pre-RNA Lipid World scenario for life's origin.

## 1. The Chemistry of Life

A central objective of any astrobiology roadmap is finding out how life began and evolved (Des Marais *et al.,* 2008; Horneck *et al.,* 2016). While the chemical paths by which life emerged are far from consensual, it is highly likely that life arose in water and that the necessary building blocks were organic compounds. Since a widely accepted definition is “life is that which replicates and evolves” (Nowak and Ohtsuki, 2008), life's origin appears to entail a transition from the abiogenic availability of various organic compounds to chemical entities capable of producing their own copies.

**FIG. 1.**
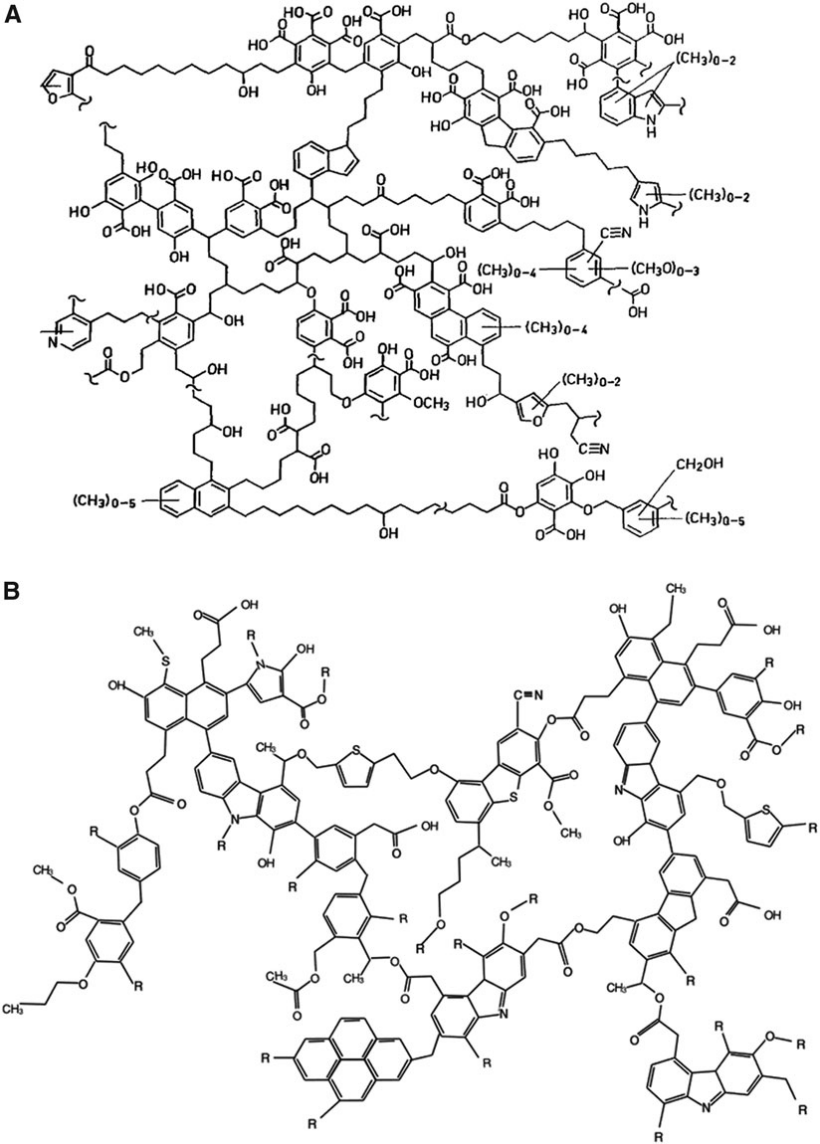
Conceptual macromolecular structures of insoluble organic matter (IOM). (**A**) Humic acid (∼3000 u) inferred on the basis of analytical degradation (Schulten and Schnitzer, 1993). Humic acid is a carbon- and oxygen-rich, largely unsaturated polymer, with an example empirical formula of C_100_H_91_O_47_N_2_S_1_P_0.05_ (Chiou *et al.,* 1987), formed upon anoxic modification of biomatter (Quentel and Filella, 2008). In some respects, it is similar to IOM in carbonaceous meteorites (Derenne and Robert, 2010) (**B**), which is less reduced (C_100_H_70_O_12_N_3_S_2_) (Postberg *et al.,* 2018). This model was built from measured elemental composition and ratios of different chemical groupings. R stands for an organic moiety. Another term used for such insoluble structures is “refractory organic material” (ROM, meaning difficult to dissolve and analyze) (Quentel and Filella, 2008). Such substances are also similar to tholins, organic heteropolymeric tarry solids (Waite *et al.,* 2007) detected, for example, in Titan's aerosols, and asphaltite-like substances observed on the asteroid Ceres (De Sanctis *et al.,* 2017). Further, similar to humic acid, the term *kerogen* is frequently used for analogous insoluble organic macromolecular materials abundant in terrestrial sedimentary rocks (Vandenbroucke and Largeau, 2007), termed also sedimentary organic matter (Leif and Simoneit, 1995). Also, the term “insoluble macromolecular organic matter” (IMOM) has been used for the product of diagenetic sequestration of microbial mat lipid biomarkers through covalent binding (Lee *et al* 2019). Finally, comparable refractory substances are generated profusely in synthetic reactions such as the Miller-Urey electrical discharge synthetic reactions (Cleaves *et al.,* 2014).

Organic compounds are those that contain covalently bound combinations of carbon with one or more of the five other main life elements, hydrogen, nitrogen, oxygen, phosphorus, and sulfur. It appears that molecular size and complexity are necessary but not sufficient conditions for being relevant to life, as some very large carbon-based molecules are abiotic (Fig. 1). Therefore, it appears that what defines life molecules is their capacity to undergo specific mutual interactions, leading to the emergence of complex networks (Toparlak and Mansy, 2018). A complementary approach has been offered (Marshall *et al.,* 2017), which aims to evaluate complex objects as possible biosignatures. The complexity measure offered (Pathway Complexity) identifies the shortest pathway to assemble an object from its basic building units.

In the time of Berzelius (1779–1848), big strides were made in studying molecules derived from living organisms, from metabolites to proteins (Kyle and Steensma, 2018). While it was clear that life molecules obeyed the rules of standard chemistry, an incapacity prevailed in synthesizing them. For the latter, Berzelius coined the term “organic” and posited that a *vital force* was necessary for their generation. A few decades later, Wöhler discovered that urea may be generated from the inorganic salt ammonium cyanate, a turning point that led to the undermining of Vitalism (Kinne-Saffran and Kinne, 1999).

Today's origin-of-life studies curiously reflect an analogous state of affairs. Contemporary laboratories can synthesize practically every molecule of life, but it appears necessary to make a distinction between organic molecules that could form abiogenically and such that could only form within evolving protocellular entities. It thus appears that our current perception of organic chemistry has shifted from a human handicap to an astrochemical one. The central challenge is deciding which organic molecules are too complex for abiogenesis, necessitating further evolution (Mann, 2012). Different origin-of-life models differ vastly in this respect.

## 2. Life's Elusive Origin: Soup or Not

**FIG. 2.**
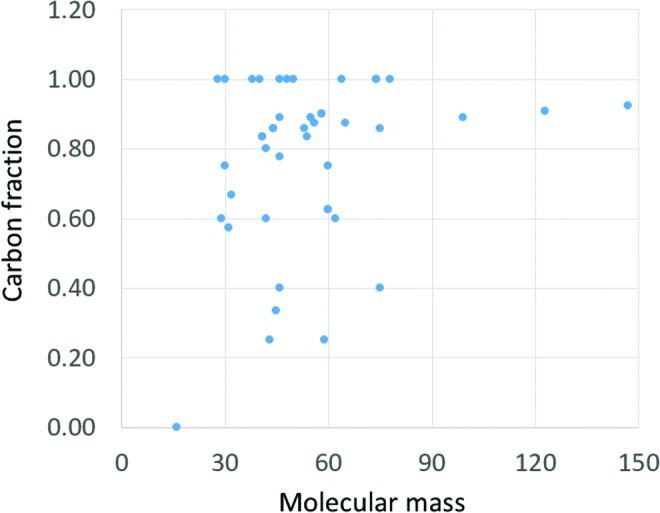
Organic molecules reported by astrochemical investigations. A collection of 53 neutral organic compounds that were detected in the interstellar medium or circumstellar shells (Klemperer, 2011) (Table 1). Topping the list are 35 compounds with 8 or more atoms (C, H, N, O, S). While some of these molecules are present in living cells, the overall picture is that of a random assortment of molecular structures, mostly with up to 7 carbon atoms. A complementary view is obtained from a recent direct analysis of comet 67P (Goesmann *et al.,* 2015), showing 12 organic compounds with up to 10 atoms (C, H, N, O), seven of which overlap with the interstellar list. Monomeric organic molecules have been detected on other planetary bodies, for example, at Gale Crater on Mars, where short aliphatic compounds up to C_5_ and sulfur-containing thiophenes have been recently reported (Eigenbrode *et al.,* 2018).

Life's origin indisputably necessitates an ample supply of organic molecules. While the origin of terrestrial organic compounds remains only partly deciphered, there is clear evidence for carbon-containing compounds wherever one probes in the Solar System. There are ample data on carbon-containing molecules up to atomic mass of 200 u in interstellar space (Fig. 2, Table 1). In icy comets, about 25% of the nucleus mass consists of carbon compounds (Greenberg, 1998). On the surface of Saturn's moon Titan there is a coexistence of organic lakes and water-ice rocks (Tokano, 2009). Based on the reported estimates of carbon infall (from dust, meteorites, and comets) during Earth's first billion years (Chyba and Sagan, 1992), one can compute that by the end of such a period the terrestrial oceans would have had an appreciable layer of insoluble organic matter in addition to ample dispersible carbon compounds.

**Table 1.** Astrochemical Molecules from Klemperer (2011)

*Name*	*Species*	*Nominal Mass*
Dihydrogen	H_2_	2
Methane	CH_4_	16
Ammonia	NH_3_	17
Water	H_2_O	18
Ethylene	C_2_H_4_	28
Methanimine	CH_2_NH	29
Carbon monoxide	CO	29
Ethane	CH_3_CH_3_	30
Nitric oxide	NO	30
Formaldehyde	H_2_CO	30
Nitroxyl	HNO	31
Methylamine	CH_3_NH_2_	31
Methanol	CH_3_OH	32
Cyclopropenylidene	c-C_3_H_2_	38
Methyl acetylene	CH_3_CCH	40
Methyl isocyanide	CH_3_NC	41
Methyl cyanide	CH_3_CN	41
Ketene	CH_2_CO	42
Cyanamide	NH_2_CN	42
Isocyanic acid	HNCO	43
Ethylene oxide	c-C_2_H_4_O	44
Acetaldehyde	CH_3_CHO	44
Formamide	NH_2_CHO	45
Thioformaldehyde	H_2_CS	46
Ethanol	CH_3_CH_2_OH	46
Dimethyl ether	CH_3_OCH_3_	46
Formic acid	HCOOH	46
Methyl mercaptan	CH_3_SH	48
Diacetylene	HC_4_H	50
Vinyl cyanide	CH_2_CHCN	53
Aluminum isocyanide	AlNC	53
Propynal	HC_2_CHO	54
Propionitrile	CH_3_CH_2_CN	55
Propenal; Acrolein	CH_2_CHCHO	56
Acetone	CH_3_COCH_3_	58
Propionaldehyde	CH_3_CH_2_CHO	58
Thioisocyanic acid	HNCS	59
Acetic acid	CH_3_COOH	60
Glycoaldehyde	CH_2_OHCHO	60
Methyl formate	HCOOCH_3_	60
Carbonyl sulfide	OCS	60
Ethylene glycol	HOCH_2_CH_2_OH	62
Methyldiacetylene	CH_3_C_4_H	64
Methylcyanoacetylene	CH_3_C_2_CN	65
Sulfur dioxide	SO_2_	66
Hexapentaenylidene	C_6_H_2_	74
Triacetylene	HC_6_H	74
Glycine	NH_2_CH_2_COOH	75
Cyanobutadiyne	HC_4_CN	75
Benzene	C_6_H_6_	78
Cyanohexatriyne	HC_6_CN	99
Cyanooctatetrayne	HC_8_CN	123
Cyanodecapentayne	HC_10_CN	147

More details in the legend to Fig. 2.

The pioneering work of Oparin (1938) provided a rational scenario, whereby abiogenic reactions and supply mechanisms delivered an abundant supply of chemical components essential for life's emergence. The organic solution that has formed in the ocean was referred to as the “Primordial Soup” or “Prebiotic Broth” (Oparin, 1962). In the century that has passed since the original Russian edition of Oparin's book, numerous scenarios have been offered regarding such supply of organic molecules. One possible path is terrestrial endogenous synthesis of organic compounds from very simple carbon-containing molecules, which necessitates a free energy source, such as light, electric discharge, or concentration gradients.

**FIG. 3.**
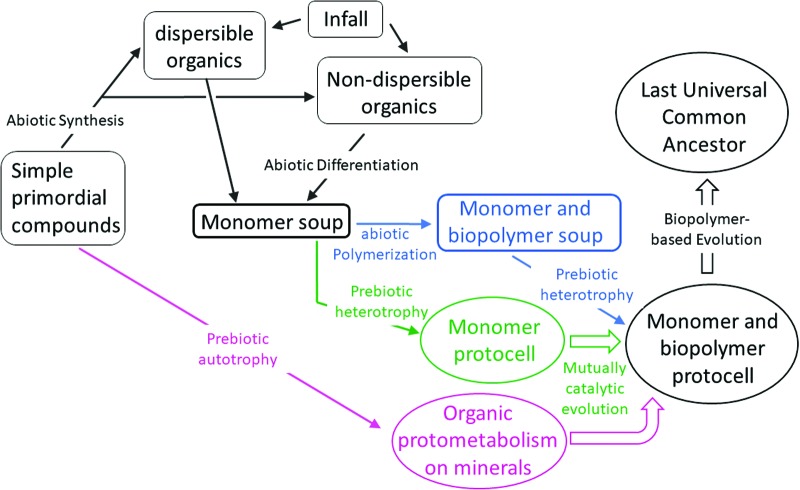
Prebiotic soup in the context of possible routes to life's origin. A prebiotic soup is by definition a water body that carries dissolved or stably dispersed organic molecules, the latter being in the form of micelles, vesicles, or emulsions. The three major sources for soup compounds are infalling water-dispersible organics, infalling water-nondispersible organics that become solubilized by abiotic differentiation, and very simple compounds that undergo abiotic synthesis to both dispersible and nondispersible compounds. The RNA World scenario calls for yet another abiotic synthesis—the generation of replicating biopolymers from specific monomers in the soup, so as to jump-start an evolutionary process (Cafferty and Hud, 2014) (blue). In contrast, the Lipid World scenario posits that replication, selection, and evolution can transpire at the level of monomers, by compositional inheritance mediated by a mutually catalytic network (Lancet *et al.,* 2018) (green). The term heterotrophy indicates that the evolving entity depends, at least partially and temporarily, on feeding on compounds from the soup. Prebiotic autotrophy (purple) is an extreme case in which the only external compounds for jump-starting life are simple molecules such as CO_2_ and water, leading to adsorbed protometabolism energized by photons, temperature or ion gradients, or mineral components (Maden, 1995). In some respects, this constitutes a flat version of a prebiotic soup (von Kiedrowski, 1996). In answer to how flat metabolism would become more life-like, the answer given is mutually catalytic evolution (Huber *et al.,* 2012), similar to what is invoked for the Lipid World.

A groundbreaking example was the Miller-Urey experiment (Miller, 1953), which demonstrated the generation of soup-worthy organic compounds from simple reducing gases energized by spark discharge. This experiment motivated several decades of scrutiny in which life-related molecules were shown to be abiotically synthesized from different precursors and based on electrical, thermal, chemical, and photochemical energy as reviewed (Ferris and Hagan, 1984; Maden, 1995; Cleaves, 2012; Bada, 2013). This mode of soup generation is represented as the abiotic path from simple primordial compounds to dispersible and nondispersible organics (Fig. 3).

While terrestrial endogenous syntheses quite strongly dominate the origin-of-life literature, exogenous infall mechanisms are at least as important (Ehrenfreund *et al.,* 2011), with sources including interplanetary dust particles, meteorites, and comets. A relevant example is the presence of dipeptides in comets, as reviewed (Kaiser *et al.,* 2013). Together, such extraneous supplies might exceed the capacity of local syntheses (Chyba and Sagan, 1992) (*cf.* Section 3). Delivered carbon compounds may also be dispersible or not; the latter referred to as insoluble organic matter (IOM, Fig. 1), which may undergo differentiation to generate smaller (monomeric) compounds with better water solubility that therefore become soup components with higher life-generation potential.

There is a faction within origin-of-life research that discounts the idea of prebiotic soup altogether (Maden, 1995). The expressed doubts go back to the Miller-Urey experiment, originally performed with hydrogen-rich reducing gases (CH_4_, NH_3_, H_2_). Subsequently, new data emerged, suggesting that at the relevant time the atmosphere was likely more oxidizing (CO_2_, CO, N_2_), making spark-induced synthesis less effective. This led to a change of mind about the existence of prebiotic soup but was later reassessed and partly reversed (Cleaves *et al.,* 2008). Notably, the soup opponents disregarded the fact that only one of many sources for soup provisions became less tenable.

Parallel support for the no-soup party came from the longstanding trophism dichotomy (Fig. 3). Two different scenarios have been invoked regarding the early steps in life's emergence. A *heterotrophic* setting assumes that emerging protolife feeds upon the organic supply in the primordial soup (Oparin and Gladilin, 1980; Bada and Lazcano, 2003). The alternative *autotrophic* setting proposed that the appearance of protocells could transpire via mineral-catalyzed thermally or photochemically energized syntheses of every organic molecule necessary, without any dependence on ready-made soup components (Hartman, 1975; Maden, 1995; Martin *et al.,* 2008; Branscomb and Russell, 2018; Kitadai *et al.,* 2018). Such a “no soup” stand has been strongly advocated based on energy and catalysis provided by the iron sulfide minerals (Wächtershäuser, 1988).

The significant doubts regarding life's origin via an autotrophic scenario (Ross, 2008; Cleaves, 2012) help keep the heterotrophic prebiotic soup concept strongly viable. The present authors are convinced that protocells with metabolism and reproduction capacities could not have emerged in water bodies totally devoid of dissolved organic compounds, as previously stated (von Kiedrowski, 1996). We posit that the invoked synthetic reactions at mineral surfaces should better be viewed as yet another soup-forming path (Gutekunst, 2018) (Fig. 3), allowing protolife to emerge heterotrophically and reach pure autotrophy at a much later evolutionary stage (Quayle and Ferenci, 1978; Maden, 1995).

The great challenge is thus to identify concrete examples of possible prebiotic soups and probe their composition. This should guide our struggle to decide how far abiogenesis can go, before the inescapable advent of reproduction-based evolution (Oparin and Gladilin, 1980). We describe here some inferences derived from the large water body on Enceladus, recently shown by direct experimental approaches to contain a diversity of organic compounds from several likely sources (Postberg *et al.,* 2018). Such direct observations, on what has been coined “the best astrobiology target in the solar system” (McKay *et al.,* 2014), constitute an excellent route to obtain hints on a possible soup composition on early Earth, and may help assess models for life's emergence out of such a soup.

## 3. The Enceladus Message

The early flybys of the Cassini–Huygens mission to the saturnian system revealed the incredible nature of Saturn's moon Enceladus. This is an active, dynamic world, including cryovolcanic plumes ejecting ice grains and vapor from faults near its south pole (Waite *et al.,* 2006; Thomas *et al.,* 2016), as reviewed (Dougherty and Spilker, 2018). Mission modifications led to Cassini flying close to Enceladus 23 times over a decade, in order to obtain more details about the moon. This established the existence on Enceladus of a 26–31 km deep global salty water ocean, which lies under a 21–26 km thick water-ice crust, with a thinner crust (≤13 km) at the south pole. The moon's center constitutes a porous rocky core of ∼200 km radius (Thomas *et al.,* 2016). The energy maintaining water in liquid state and driving the plumes' eruptions is likely derived from tidal dissipation (Nimmo *et al.,* 2014).

**FIG. 4.**
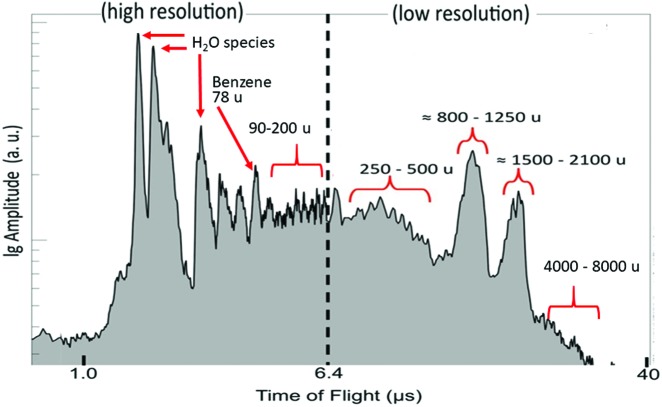
Mass spectrum from the Cassini Cosmic Dust Analyzer (CDA), adapted from extended data Fig. 5A in Postberg *et al.* (2018). More details are in the text.

Early chemical analyses of the plumes, and of Saturn's E-ring generated from their ejecta, established compositions of organic molecules in an H_2_O-dominated context (Waite *et al.,* 2006; Postberg *et al.,* 2008, 2011). These observations suggested organic compounds with 2–4 carbon atoms, potentially derived from larger aromatic entities. A recent publication (Postberg *et al.,* 2018) has extended these observations, providing a much more detailed picture of the enceladan ocean's organic repertoire (Fig. 4). The new data suggest that some of the ice grains contain concentrated and much more complex organic material. Using their nominal sensitivity range, we found that the mass spectrometry observations extend the organics' size range from <50 u (atomic mass units) to ≤200 u (Fig. 4). This higher range, defined as high-mass organic cations (HMOCs), likely represents molecules with 7–15 carbon atoms.

Extended mass spectra (beyond the nominal range of the Cassini instrument) exhibited indication for yet larger molecules, with atomic masses up to ∼8000 u, with prominent propensity peaks at 200–300, 800–1100, and 1600–1800 u (Fig. 4). Further support for such large molecules is obtained by inference based on the relative intensities of the increasingly heavy HMOC peaks at <200 u, and on their dependence on grain impact speed, suggesting that HMOCs are fragments of higher-mass parent macromolecules, with up to 150 carbon atoms (2000 u).

The very large molecules are inferred, based on interpeak spacing and carbon to hydrogen ratio of ∼1, to be composed of polymerized isolated aromatic rings, along with unsaturated aliphatic links, potentially with functional groups containing oxygen and probably nitrogen. A lower likelihood is ascribed to the possibility that the detected macromolecules constitute fused-rings polycyclic aromatic hydrocarbons (PAHs), which would show higher carbon to hydrogen ratio. The present authors suggest that the macromolecular compounds detected in the enceladan plumes at the heavier end of the spectrum are related to complex molecules with a web of mutual covalent links (Fig. 1).

## 4. Enceladus' Primordial Soup

The discovery of diverse organic compounds within a substantial water body on a Solar System moon allows one to obtain new information regarding possible conditions on early Earth. The enceladan subglacial ocean appears to be the first observationally discovered concrete example of what may well be a primeval soup. We note that this statement could be hampered if some forms of life exist now or existed earlier in the enceladan ocean. This scenario could be examined, for example, if a more detailed analysis of amphiphiles embodied in the enceladan organic matter showed correspondence to the characteristic lipid pattern expected from a biological process (Georgiou and Deamer, 2014). However, in the absence of evidence to the contrary, a biotic origin of Enceladus' organics remains a remote possibility (Postberg *et al.,* 2018).

What Cassini has discovered in the plumes must reflect the chemical and physical conditions of the subsurface ocean and its dynamics over time. In particular, one is curious about the organic molecules that are dissolved, dispersed, or suspended in this aqueous body. The power generated in the core from tidal friction creates high temperatures and thermal gradients in the ocean (Choblet *et al.,* 2017). Further, silicon-rich nanoparticles observed in the plumes have suggested the existence of active hydrothermal vents at the water-rock interface, where temperatures are about 90°C and alkaline pH of 8.5–10.5 and pressures of 10–80 MPa prevail (Hsu *et al.,* 2015). Temperatures inside the porous core, where hydrothermal activity is initiated, may be much higher (Travis and Schubert, 2015).

Organic layers, whether liquid oils floating on the ocean's surface (Postberg *et al.,* 2018) or organic solids sinking to the rocky core, would undergo chemical alteration and differentiation, interacting with core minerals and metal ions to generate new chemical compositions. Molecular hydrogen, detected in the plumes and likely reflecting hydrothermal activity at the core (Waite *et al.,* 2017), along with other reducing gases such as methane and ammonia could contribute to further chemical modifications.

The plume analytics in all probability should represent such cross-oceanic chemistry based on the reported upward transportation having a timescale of years (Sekine *et al.,* 2015). The cumulative evidence from the enceladan ocean thus suggests a chemically rich and physically dynamic water body, as befits a prebiotic soup (Wollrab *et al.,* 2016). Further, that the enceladan ocean resides under a thick layer of ice and still becomes rich in increasingly complex organic compounds opposes the notion that atmospheric sparking is crucial for the emergence of a prebiotic soup (Maden, 1995). We note, however, that alternative free radical reactions could supplement this missing synthetic pathway.

## 5. Monomers and Polymers

How fully do the instrumental observations from Enceladus reflect the potential chemical diversity of the subglacial ocean? Regrettably, the resolution of Cassini's instrumentation rarely allows one to identify individual organic molecules in the plume ice grains. Judging merely by size, the lower mass range could represent diverse bio-monomers, such as amino acids (75–200 u), nucleosides (100–300 u) and sugars (200–400 u). However, chemical scrutiny indicates that the molecular properties of the enceladan compounds are quite different from those of most biomolecules. First, the abundance of heteroatoms (oxygen and nitrogen) appears low, similar to that of primordially accreted organic matter (O/C ≈ 0.2, N/C ≈ 0.04) (Alexander *et al.,* 2017). On the other hand, the observations are consistent with a predominance of relatively long unsaturated hydrocarbon chains, aliphatic or aromatic, with a small number of polar heteroatoms. These molecular characteristics are reminiscent of those affiliated with amphiphilic compounds. We note that while most present-day cellular lipids have aliphatic hydrocarbon chains (Georgiou and Deamer, 2014), there are reported instances of lipids with aromatic hydrophobic moieties (Bhattacharya and Biswas, 2010).

The higher mass spectrometry fraction (1000–8000 u) spans a range akin to oligomeric or polymeric macromolecules. By size, these could represent linear biopolymers, for example 10–70 amino acid long polypeptides, or a 2–16 long oligonucleotide or 3–40 long oligosaccharide. However, there are counter-indications that make this hypothesis untenable, including the CH-rich, aromatic nature and apparent water insolubility of the observed high molecular mass organics, for which the more probable candidates are kerogen-like compounds (Postberg *et al.,* 2018).

The Enceladus mass spectrometry measurements have left it open whether the organic compounds in the plumes actually include monomers (<200 u). As mentioned (Section 4), it is possible that the low-mass-range molecules are artifactual fragmentation products of the higher-mass substances. If so, what was detected in the plumes contains majorly insoluble polymeric substances. But this does not mean that the moon's ocean would not contain smaller molecules. It is possible that the saltwater grains contain monomeric organic material, which stays below the sensitivity threshold of the Cassini instruments. Future missions might be able to characterize such organic molecules, by extending the geophysical characterization toward the search for life-worthy organic molecules (McKay *et al.,* 2014). We note that another set of Cassini results was obtained near Saturn's moon Titan, in which the atmosphere was scrutinized by mass spectrometry without the involvement of ice grains (Waite *et al.,* 2007). This shows a very similar picture to that reported for Enceladus, that is, carbon compounds in the monomer range (80–350 u) as well as polymers sized up to 8000 u, perhaps pointing to bona fide organic monomers in Titan's atmosphere.

## 6. Toward Life

It is important to assess the capacity of Enceladus' aqueous organic content to jump-start life. This complements other legitimate questions regarding the similarity of the moon's environments to those proposed by some as essential for terrestrial life's origin—hydrothermal vents and volcanic fields (Deamer and Damer, 2017). Obviously, kerogen-like water-insoluble organic polymers would be judged by many as far from optimal for life's emergence. Therefore, one should explore the possible chemical paths by which smaller molecules would coexist with, or be generated from, the water-insoluble mass.

In this realm, considerable insight may be obtained from the experiments performed on carbonaceous meteorites (Cronin and Chang, 1993; Pizzarello and Shock, 2017). This is a group of asteroid fragments containing between 2% and 8% carbon (Kerridge, 1985), including kerogen-like macromolecules (Fig. 1) (Kerridge *et al.,* 1987), as well as numerous simpler compounds (Cronin, 1998). They therefore may carry a record of early carbon chemistry, and provide clues to the organic compounds on planetary and subplanetary bodies, including on ocean worlds, pointing to the compositions that might have led to the onset of life (Pizzarello and Shock, 2017). Such an approach is vindicated by published statements that the composition of Enceladus' core bears similarity to that of carbonaceous chondrites (Sekine *et al.,* 2015; Postberg *et al.,* 2018).

**FIG. 5.**
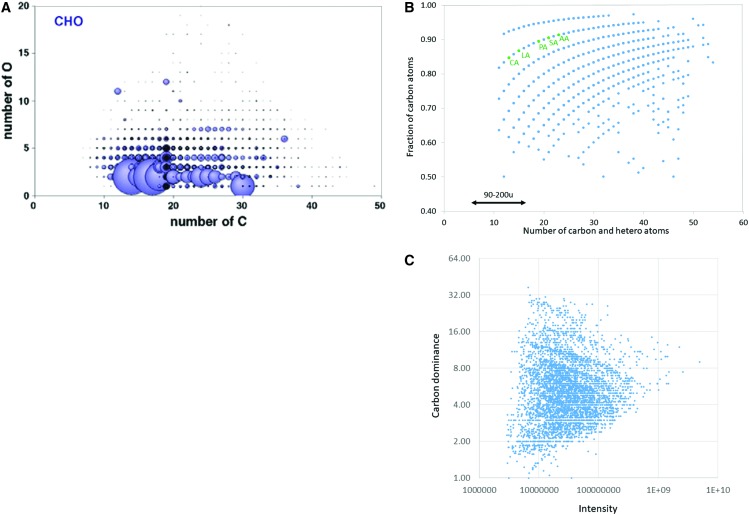
(**A**) Distribution of mass spectrometry peaks in extracts of a Murchison meteorite sample. Shown here are the molecular series of compounds containing only carbon, hydrogen, and oxygen showing the number of oxygen atoms (vertical axis) versus carbon atoms (horizontal axis). Circle areas are related to mass peak intensities; here, singular large peaks likely denote terrestrial contamination. The bulk of the compounds are C_12_–C_30_ with the main intensities in the O_2_ row, consistent with amphiphilic character. Adapted from Fig. S8A in Schmitt-Kopplin *et al.* (2010). (**B**) Carbon atom enrichment distribution of non-sulfuric compounds extracted by methanol from a Murchison meteorite sample. Molecules labeled in green are graph positions consistent with fatty acid lipids: CA, capric acid (C_10_); LA, lauric acid (C_12_); PA, palmitic acid (C_16_); SA, stearic acid (C_18_); AA, arachidic acid (C_20_). The double arrow indicates the approximate molecular mass range of the heavy species seen in the high-resolution part of Fig. 4. Thus, the entire range of this monomeric collection spans up to ∼700 u. (**C**) Intensity distribution of the Murchison compounds shown in (B). It is apparent that in the main the compounds span 1.5 orders of magnitude of concentrations.

Earlier analyses of the Murchison carbonaceous meteorite showed that its organic material is largely macromolecular. But in focused analysis aimed at lifelike small molecules, extracts were found to contain a complex mixture of hundreds of monomeric compounds, including carboxylic acids, amino acids, hydroxy acids, phosphonic acids, amines, amides, nitrogen heterocycles (including purines and pyrimidines), alcohols, as well as aliphatic, aromatic, and polar hydrocarbons (Cronin and Chang, 1993; Cronin, 1998). A more recent paper (Schmitt-Kopplin *et al.,* 2010) provided a nontargeted high-resolution molecular analysis of the solvent-accessible compounds. The results portray ∼30,000 different molecular compositions sized 100–1000 u, each being a compound set sharing an empirical formula. Thus, the meteorite appears to contain millions of specific molecular structures yet to be fully resolved. An example analysis indicated that among methanol-soluble compounds with only C, H, and O atoms, the great majority have 12–30 carbons and 1–2 oxygens, attesting to a likely preponderance of amphiphiles (Fig. 5A). Another analysis that includes all compounds with C, H, N, and O atoms (Fig. 5B), shows empirical formulae corresponding to specific fatty acid lipids. Such data lend strong support to the earlier observation that components in a Murchison extract form vesicular boundary structures (Deamer, 1985). One could thus hypothesize that future detailed analyses of Enceladus' insoluble organic polymers could reveal similar chemical constituents, stemming either from a possible native heteroatom content of the insoluble matter, as in humic substance and in the Murchison meteorite (Fig. 1), or from hydrolysis (see below).

In parallel, high-temperature hydrous pyrolysis experiments of solid fossil fuels such as kerogen and bitumen, in the presence or absence of mineral catalysts, gave rise to a plethora of low-molecular-weight organics. The products included alkanes, alkenes, and isoprenoids (Huizinga *et al.,* 1987) as well as long-chain fatty acids (Kawamura *et al.,* 1986; Siskin and Katritzky, 1991). In another set of studies, hydrous pyrolytic degradation of petroleum sediments (kerogen) has been examined both in hydrothermal vents and in the laboratory (Leif and Simoneit, 1995). A major set of products constituted amphiphilic n-alkanones with chain lengths from C_11_ to C_33_. A similar set of results showed how hydrothermal pyrolysis leads to the formation of diverse polar compounds, including alkanoic acids and alcohols, isoprenoid ketones, and alkanoate esters, in the C_9_–C_33_ range (Rushdi and Simoneit, 2011). All these results are consistent with the possible generation of monomeric organics from insoluble polymers under conditions similar to those prevailing on Enceladus. Whether this actually happens on Enceladus could only be verified by future missions and analyses.

In the above portrayals, many of the monomeric compounds are of low polarity. Would such molecules become water-dispersed in an Enceladus-like ocean, and in sufficiently high concentrations to afford the formation of a prebiotic soup? One relevant fact is that H_2_O at high temperatures becomes a much better solvent for organic molecules (Siskin and Katritzky, 1991). In parallel, it is noteworthy that amphiphiles, prominently featured in the above studies, naturally aggregate to form micelles and vesicles, thereby greatly enhancing their dispersal capacity. Thus, while a C_16_ fatty acid lipid has a ∼1 nM monomeric solubility (Smith and Tanford, 1972), it can easily reach a micellar concentration × 10,000 higher before it approaches the point of phase change (Lobry *et al.,* 1999). Along similar lines, the Enceladus report (Postberg *et al.,* 2018) suggests the formation of amphiphilic micelles upon water-mediated oxygenation of the reported plume macromolecules.

Further, micelles of amphiphilic compounds help solubilize otherwise water-insoluble hydrophobic compounds, absorbing them into their lipophilic cores (Bissette *et al.,* 2014). At higher absorption stoichiometry, structures with larger hydrophobic cores emerge, such as nano- and micro-emulsion droplets (Kronberg *et al.,* 2014; Kampmann *et al.,* 2016), thus increasing the aqueous concentrations of the water-insoluble compounds 1,000-fold (Uchegbu, 2013). In the same vein, it has been proposed that insoluble disordered organic compounds (tars) could be incorporated into ordered lipid bilayer structures (Yeom *et al.,* 1995; Bywater, 2012). Importantly, micelles may also catalyze the covalent transformation of such absorbed hydrophobic molecules to generate additional amphiphiles (Post *et al.,* 2018).

**FIG. 6.**
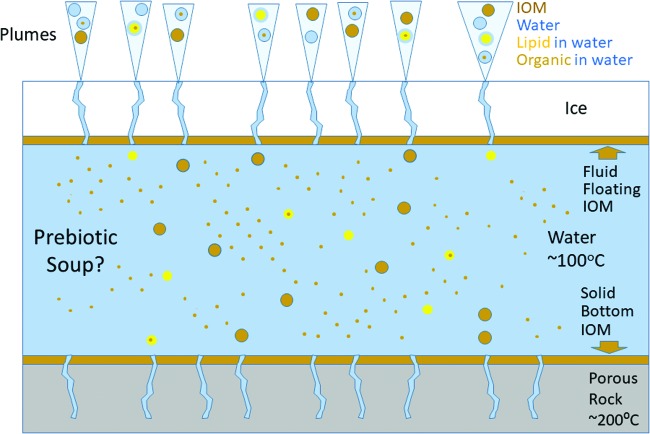
Enceladus cross section, showing the different potential components of the soup and the plumes. IOM stands for insoluble organic matter, which is taken to be synonymous with nondispersible organic matter in Fig. 3. Such polymeric compounds can still be carried in the plume as small particles detached from insoluble organic layers at the top of the water layer. Lipid in water alludes to monomers and aggregates such as micelles and vesicles. Organic in water is dispersed, for example as microemulsion. Inspired by the data in Postberg *et al.* (2018).

In sum, the enceladan apparent soup may well have a significant and diverse organic repertoire. It may be profusely populated with dispersible aggregates containing both amphiphiles and hydrophobic compounds (Fig. 6), bearing significant resemblance to Oparin's originally invoked colloidal coacervates (Oparin, 1938; Oparin and Gladilin, 1980). The exact constituents and relative amounts are presently unknown, but such information could be gleaned by further analyses of the Cassini results and by carefully crafted future missions, equipped with capacities to identify individual monomeric species.

## 7. Prioritizing Origin Scenarios

In an insightful review (Chang, 1993), the author asks a fundamental question: What organic compounds were required for the origin of life? He points out that historically the answer has been shaped by a scientist's favorable model: “Metabolism First” calls for carbohydrates, “Protein First” assigns a key role to amino acids, “Lipids First” claims a major involvement of compartment-forming amphiphiles, and “RNA First” advocates the primacy of nucleic acids. The author summarizes this essential incongruity by pointing out that practically all answers are molded by predictions on what early life would have looked like, rather than by consideration of the actual environments in which life may have arisen.

Thus, Chang suggests that any discussion of life's origin should reflect a planetary environmental perspective. Accordingly, we focus here on asking which emergence-of-life scenario is most compatible with the best predictions regarding the organic content of actual prebiotic water bodies. In doing so, it seems appropriate to focus on the likely monomeric compounds as a basis for prioritizing origin-of-life hypotheses. This is because it is more difficult to envisage life emerging directly from simple gases or from random polymers: life's common origin must at one point pass through biochemistry-like mutual interactions of medium-sized molecules such as amino acids, nucleotides, and lipids (Patel *et al.,* 2015).

The most prominent characteristic of the astrobiological monomer repertoire as described above is its molecular diversity. Carbonaceous meteorites contain at least 30,000 monomer types with carbon atom counts of 6–37 and with a relatively narrow abundance range (Schmitt-Kopplin *et al.,* 2010) (Fig. 5C). Similar monomer diversity arises in Miller-type synthesis with mixtures of gases (Wollrab *et al.,* 2016). The authors note: “The capacity to spontaneously produce this extremely high degree of molecular variety in a very simple experiment is a remarkable feature of organic chemistry and possibly prerequisite for life to emerge. It remains a future task to uncover how dedicated, organized chemical reaction pathways may have arisen from this degree of complexity” (Wollrab *et al.,* 2016).

The scenarios of Metabolism First, Protein First, and RNA First require very specific monomers, which may well be represented in the soup at <1/1000 fractions. The enormous chemical competition from “nonbiological” monomers would make the probability of biopolymer synthesis from such monomer mixtures vanishingly small (Shapiro, 2000). It thus becomes advisable to explore an origin scenario that fulfills the following criteria:

(a)  Unexclusive involvement of present-life-like monomers and polymers
(b)  Consistency with large monomer diversity
(c)  Consistency with and benefitting from high abundance of carbon-rich compounds
(d)  Compatibility with low organic compounds concentrations
(e)  Compatibility with extreme conditions
(f)  Independence of an external energy source

As befits a good scientific model, many of these criteria are related to a single chemical phenomenon, namely hydrophobic interactions, as portrayed by Tanford (1978). But rewardingly, the entire set of criteria results in a chemical gestalt that helps prioritize origin models.

It appears that among the major origin scenarios one stands out as having a higher conformity with the above set of criteria. This is the Lipid World scenario, as described by Segré *et al.* (2001a,b) and Lancet *et al.* (2018). In its full-fledged manifestation, it goes beyond proposing that lipids were essential for encapsulating informational molecules and catalysts (Deamer, 2017). Rather, it posits that the first replicators were lipid assemblies (most likely micelles), capable of reproducing their compositional information, driven by non-enzymatic catalysis in the realm of mutually catalytic networks (Lancet *et al.,* 2018). It is further suggested that the advent of life-like nucleic acids, proteins, and metabolites was the result of a prolonged evolutionary progression of reproducing amphiphile assemblies, which gradually developed a capacity to absorb or endogenously synthesize the necessary heteroatom-rich monomers.

**FIG. 7.**
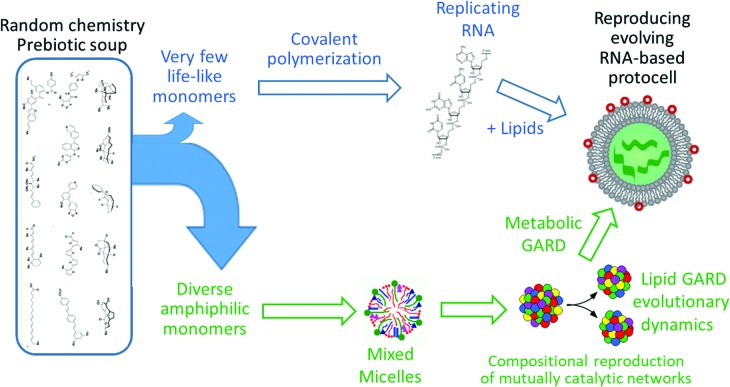
From soup to protocells, delineating the underpinnings of two different models, RNA First (RF, top) and Lipid First (LF, bottom). In RF, specific monomers are singled out from a heterogeneous chemical mixture and undergo polymerization, culminating in the emergence of a self-replicating polymer. This is then enclosed in a lipid bilayer, leading to protocells capable of selection and evolution. In LF, a large variety of amphiphiles spontaneously generates a plethora of assemblies, for example micelles. The GARD model then predicts that very specific micellar compositions establish a mutually catalytic network, which may exhibit homeostatic growth. This, when followed by fission events, constitutes a reproduction system, capable of selection and evolution (see “How do lipids reproduce?” Section 8.4 and Lancet *et al.* [2018]). Subsequent prolonged evolution may lead to the emergence of RNA, proteins, and metabolism (See Section 8.5, “How would lipids beget full-fledged protocells” and Lancet *et al.* [2018] Section 11.1). The pros and cons of the two models are summarized herein:
(i)Compatibility with the chemical diversity: RF depends on selection or synthesis of a very specific subset of monomeric compounds, such as four nucleotide bases and the sugar ribose. Chemical diversity is an impediment, leading to side reactions and making the spontaneous emergence of nucleotides and their linear polymer less likely (Shapiro, 2000). LF is promiscuous, with amphiphile assemblies readily emerging out of a very complex monomer mixture.(ii) Facility of chemical reactions: RF requires covalent polymerization, necessitating activated monomers as a free energy source. LF stays away from equilibrium by spontaneous “noncovalent polymerization” toward micelle formation, an energetic downhill reaction driven by hydrophobic interactions, and via physical disruption that leads to fission.(iii) Need for a concentration mechanism: In RF, for a biopolymer to form, a monomer concentration mechanism is needed. This is assumed to happen by an extraneous agent, such as heat-induced drying, absorption on mineral surface, or restraining within lipid vesicles or mineral pores. LF has a built-in concentration mechanism, based on the hydrophobic interactions among tails, which bring the headgroups together.(iv) Heat stability: In RF, the biopolymers are heat labile, in terms of both polymer covalent integrity and three-dimensional structure (Shapiro, 2000). In LF, amphiphile assemblies are considerably more heat stable, with hydrophobic interactions augmented with increasing temperature.(v)Information content: For replication/reproduction, information has to be stored and transmitted across generations. The sequence-based information in RF is considerably more effective and has the advantage of being copied through a base-pairing mechanism. In LF, compositional information, although less efficient, is still stored and transmitted upon assembly growth-fission cycles. While there is an admitted paucity of experimental data to directly back such a scenario, supporting data are beginning to accumulate (Bukhryakov *et al.,* 2015), and it is hard to miss the analogy of compositional reproduction to epigenetic inheritance (Pulselli *et al.,* 2009), a cornerstone of information transfer in nowadays living cells (Dupont *et al.,* 2009).(vi) Catalysis: In parallel to free energy source, this is a fundamental requirement for a lifelike entity to sustain itself away from equilibrium. Enhanced catalytic capacities are also crucial for transiting from a “naked replicator” to a protocell endowed with metabolism and genetic encoding. Regarding RF, there is a large body of experimental evidence for RNA being catalytic, including rate enhancement of biopolymer formation and of metabolism-like reactions. For LF, direct evidence is less abundant, but as amphiphiles are flexible in headgroup choice, amino acids or peptides may play this role, conferring catalytic properties (Zhang, 2012). In addition, micelles may act catalytically to make more amphiphiles as experimentally documented (Bachmann *et al.,* 1992; Bissette *et al.,* 2014; Post *et al.,* 2018). Catalysis is facilitated by restriction to 2-D diffusion (reduced dimensionality [McCloskey and Poo, 1986]) and by curvature-induced free energy gradients (Seifert, 1993). Lipid assemblies have also been experimentally shown to harbor rudimentary mutually catalytic networks, with a capacity for replication-like behavior (Bukhryakov *et al.,* 2015; Hardy *et al.,* 2015).

In more detail, the Lipid World scenario, embodied in the Graded Autocatalysis Replication Domain (GARD) model, fulfills all the above-mentioned needed criteria as follows (Lancet *et al.,* 2018) (see Fig. 7 for additional details and comparison to the RNA World model):

(a)  The GARD model is opportunistic: it does not necessarily require the availability of specific molecular types, not even specific present-life lipids, such as phospholipids, sphingolipids, and cholesterols. Rather, any amphiphile with the right physicochemical properties is legitimate.
(b)  A prerequisite of the GARD model is the availability of a highly diverse repertoire of lipid-like molecules, which generate multitudes of assemblies with different molecular compositions. Kinetic computer simulations demonstrate that some such assemblies will have a capacity to reproduce by homeostatic growth followed by random splits, a dynamic state called composome (Segré *et al.,* 2000).
(c)  Simple amphiphiles are carbon-rich by nature, typically having 10–30 carbon atoms in their hydrophobic tail (or tails), and very few (1–3) heteroatoms.
(d)  Because of spontaneous assembly formation, for example micelles, lipid-like molecules can undergo effective mutual interactions. Critical micelle concentration values for different lipids cover a wide range between 10 mM and 1 nM (Marsh, 2013), with lower values for compounds with longer chains and/or 2 acyl chains. Thus, under prebiotic conditions micelles are likely to form for at least some of the amphiphilic soup components, even if present at rather low concentrations, and without a need for drying processes.
(e)  Lipid assemblies are heat resistant, as they form via hydrophobic interactions, which increase in strength with elevated temperatures (van Dijk *et al.,* 2015). Such assemblies also withstand extreme pH (Otzen and Andersen, 2013).
(f)  While other models require an external energy supply (activated monomers) for covalent polymerization, GARD feeds on endogenous energy—the formation of micelle (noncovalent “polymers”) driven by hydrophobic interactions is naturally downhill in terms of free energy change.

It is still necessary to ask whether the enceladan ocean environment is conducive to a Lipid First scenario. When judging such a scenario in terms of the formation of cell-like containers, it is necessary to take into account parameters such as amphiphile concentration and structure, temperature, mineral involvement, salt concentrations, and pH ranges (Deamer and Georgiou, 2015). We, however, note that a scenario such as GARD is less sensitive to many of these parameters, because in its early stages it does not necessitate an aqueous lumenal enclosure within a stable bilayer.

## 8. Can Lipids Beget Life?

Some seminal questions are often posed regarding the validity of the Lipid World scenario. These are briefly addressed below and in considerably more detail in Lancet *et al.* (2018). The main questions are as follows.

### 8.1. Where do lipids come from prebiotically?

A rather detailed answer is provided in the foregoing Sections 6 and 7, addressing the high probability of diverse primordial and alteration-produced amphiphiles. In parallel, the abiotic synthesis of amphiphiles from simple compounds has been demonstrated, as exemplified by the formation of lipids via high-temperature aqueous Fischer-Tropsch reaction from oxalic acid (Rushdi and Simoneit, 2001).

### 8.2. Can lipids be catalytic?

A centerpiece of the GARD model, as established nearly 20 years ago (Segré *et al.,* 2000), is the capacity of lipids to exert catalysis. Since then this notion has been further elaborated in detail in a large number of papers from our laboratory, as exemplified (Segré and Lancet, 2000; Segre *et al.,* 2001a,b; Shenhav *et al.,* 2003, 2004, 2005; Bar-Even *et al.,* 2004; Markovitch and Lancet, 2012, 2014; Gross *et al.,* 2014). A detailed review on the realism of lipid catalysis, including entire monographs on lipid and micellar catalysis (Fendler and Fendler, 1975; Fendler, 1982), has recently been published (Lancet *et al.,* 2018, Section 10.1). The foregoing body of knowledge is consistent with the proposition that early on, in the likely absence of enzymes, lipids might have fulfilled rate enhancement functions. This was made possible by non-enzymatic catalysis, a property manifested by a large variety of chemical groupings (Fendler, 1982; Segré *et al.,* 2001a; Wallace and Balskus, 2014; Lancet *et al.,* 2018). Further, because early lipids were unconstrained in their chemical configurations, some could include well-established catalysts as headgroups, including peptides (Zhang, 2012) and metal chelating ligands (Hardy *et al.,* 2015). Finally, lipid aggregates constitute a reasonable alternative to catalytic mineral surfaces (Sargent and Schwyzer, 1986; Bissette *et al.,* 2014; Ortega-Arroyo *et al.,* 2016).

### 8.3. Can lipids store and propagate information?

The GARD model is based on the storage and transmission of compositional information, which is different from but equivalent to sequence-based information. In present-day life, living cells manage sequence information mediated by polynucleotides as well as compositional information, which is stored and reproduced via what is known as epigenetic inheritance (Dupont *et al.,* 2009). The Lipid World is hypothesized to initially rest on pure compositional information, and the GARD model portrays in detail how this may have transpired (Segré *et al.,* 2000, 2001a,b; Gross *et al.,* 2014; Markovitch and Lancet, 2014).

### 8.4. How do lipids reproduce?

The replication (or reproduction) of lipids is a property associated with an entire assembly of amphiphilic monomers, consistent with the notions of mutually catalytic networks (Kauffman, 1986; Hordijk *et al.,* 2010) and systems protobiology (Lancet *et al.,* 2018). GARD invokes a chemical process that constitutes growth by monomer addition, directed by a mutually catalytic network from within the assembly. Computer simulations show that privileged lipid compositions (composomes) undergo homeostatic (concentration-preserving) growth. Such growth constitutes the essence of a reproduction capacity, much like what happens in growing cells when they expand while their internal metabolic networks keep the ratios among all molecular constituents largely unchanged. As in living cells, subsequent assembly fission generates two similar progeny, a last step in the reproduction sequence (Segré and Lancet, 2000). The notion of compositional homeostasis as a proxy for reproduction is elaborated in greater detail in Lancet *et al.,* 2018, Section 3).

### 8.5. How would lipids beget full-fledged protocells, including metabolism, RNA, and proteins?

We note that the same question may be asked about naked RNA replicators, with respect to acquiring metabolism, lipids, and proteins. The advantage of Lipid First assemblies is that being capable of systems reproduction, selection, and evolution (Markovitch and Lancet, 2014), they can complexify by evolutionary paths simulatable by molecular dynamics (Lancet *et al.,* 2018). Since lipids with peptide and oligonucleotide headgroups do exist (Bukhryakov *et al.,* 2015; Hardy *et al.,* 2015), new chemistries can emerge, based on heterotrophic availability or endogenous evolution-driven synthesis. Notably, since lipids in present-day life obey specific structural and functional constraints (Georgiou and Deamer, 2014), lipid structure evolution is also expected to occur en route to more elaborate life-forms.

## 9. Conclusions

A prebiotic soup is any water body containing organic compounds from a variety of supply sources. Arguments against the existence of a prebiotic soup are not strong enough to negate its likely existence and role in life's origin. The Enceladus observations teach us by direct measurements that an extraterrestrial organic-rich soup exists even in the absence of an atmosphere. Such a soup is likely fed by primordial accretion, infall, and subsequent abiotic chemical modifications and syntheses, the combination of which may increase its diversity.

The Enceladus data are consistent with a soup replete with highly diverse organic compounds, which could serve as chemical supply for the formation of early lifelike entities. Abiogenesis and further evolution would necessarily occur in a heterotrophic path, until the much later advent of cellular autotrophy. The suitability of a model for life's origin would depend on the degree to which it fits the inferred soup composition, including supplementation by widely published paths for the abiotic synthesis of lifelike molecules (Cafferty and Hud, 2014). This review addresses a possible parsimonious scene, whereby the advent of very early life may have depended chiefly on the components of the non-supplemented Enceladus-like soup, and that this course fits well with our Lipid First GARD model.

We point to several characteristics of the GARD Lipid World scenario, which are highly compatible with Enceladus-style soup. First, GARD is consistent with, and in fact benefits from, a soup's large molecular diversity. Second, the moon's chemistry is consistent with considerable amphiphilic abundance. Third, GARD is compatible with low concentrations of organic compounds. Fourth, while other models require an external energy supply, GARD feeds on endogenous energy. And fifth, lipid assemblies are much more resistant to extreme conditions. We further note that the GARD model affords a gradual simulatable path from Lipid World to life as we know it, turning such emergence into a problem in evolutionary dynamics.

Our comparison between the RNA First and Lipid First scenarios (Fig. 7) is not intended to negate the crucial role of RNA in life's emergence. Rather, it points to the possibility that Lipid First non-equilibrium, catalysis-based dynamics could serve as a pre-RNA first step on a long road to full-fledged protocells. Such portrayal addresses the widely expressed doubts regarding the probability of molecules as complex as self-copying covalently strung RNA to emerge directly from random chemical mixtures (Shapiro, 2006). We suggest that such direct emergence may be more probable for a much simpler system, a self-copying noncovalent assembly of catalytically interacting amphiphiles. This statement is backed by explicit computations of the probability of appearance, on a planetary scale, of self-reproducing lipid micelles (Lancet *et al.,* 2018, Section 13).

A nagging question is which of the two scenarios is more probable, when assessing the transition from an initial replicator to a full-fledged protocell with RNA, proteins, membranes, and metabolism. This transition is considered by some to be on the verge of magic (Ralser, 2018). In this context, RNA First proponents use statements such as “The first … [entities] are RNA replicase and a self-replicating vesicle, [which later] combined into a protocell, enabling rapid evolutionary optimization” (Szostak *et al.,* 2001). Similarly, it is stated, “[at] first … RNA was the only component of biological catalysts, the second episode began with the invention of translation-based synthesis of proteins” (Benner *et al.,* 1989). These portrayals do not specify a molecular mechanism for the major transitions involved. The key difficulty is that, in an RNA-centric view, evolution cannot begin before several life component types join forces. An advantage of the GARD Lipid World view is that rudimentary evolution becomes possible at the lipid-only stage, and RNA, proteins, and metabolism may then emerge in the realm of a long, gradual Darwinian evolutionary process, in a set of molecularly describable routes (Lancet *et al.,* 2018, and Fig. 7).

The Enceladus analyses do not constitute a factual proof for the Lipid First scenario; they only make it more likely in terms of building block availability. Thus, at present, it is hard to reach a final verdict regarding an overall favorable scenario for life's origin. The idea of compositionally rooted lipid-based origin may therefore offer important though unorthodox goals for planetary missions. A focus on sequence-encoded information calls for seeking linear polyelectrolytes (Benner, 2017). In contrast, the lipid composition scenario suggests a quest for early assemblies of more apolar and smaller molecules. A higher mass-spectrometry resolution could reveal the predicted lower-mass building blocks and their diversity. It may also be able to detect narrower chemical repertoires in molecular assemblies as compared to that of their environment, as predicted (McKay *et al.,* 1996; Lancet *et al.,* 2018). These further points of evidence could be of help when seeking evolving entities in the enceladan ocean and elsewhere. In sum, more extensive evidence from astrobiological research, chemical experimentation, and computer analyses would be necessary for the ultimate arbitration between origin-of-life scenarios.

## Abbreviations Used

GARD: Graded Autocatalysis Replication Domain
HMOCs: high-mass organic cations
IOM: insoluble organic matter
u: atomic mass units
